# Lung particle overload: old school –new insights?

**DOI:** 10.1186/s12989-015-0086-4

**Published:** 2015-04-26

**Authors:** Paul Borm, Flemming R Cassee, Günter Oberdörster

**Affiliations:** Zuyd Hogeschool, Department of Life Sciences & Health, PO Box 550, 6400 AN Heerlen, The Netherlands; National Institute for Public Health and the Environment (RIVM), PO Box 1, 3720 BA Bilthoven, The Netherlands; Institute of Risk Assessment Sciences, Utrecht University, PO Box 80.163, 3508 TD Utrecht, The Netherlands; Professor Emeritus of Toxicology, University of Rochester, Department of Environmental Medicine, Rochester, NY 14642 USA

It is more than 25 years ago that the late Paul Morrow published his paper entitled “Possible mechanisms to explain dust overloading of the lungs” [1]. The paper had a major impact on our thinking on the retained dose and clearance upon inhalation of respirable dust. We have to realize that in those years’ asbestos, man-made mineral fibres and coal-mine dust induced pulmonary diseases dominated the publications. Particle and Fibre Toxicology was not yet started, and fundamental discussions on general particle paradigms were disseminated through Inhalation Toxicology and general Toxicology journals, such as the journal chosen by Paul Morrow, Fundamental and Applied Toxicology. Ambient fine particles and nanoparticles were not yet explored by particle toxicologists, [which is hard to imagine] in stark contrast to these days, where 90% of papers in Particle and Fibre Toxicology focus on nanomaterials, and fundamental mechanisms of particle induced cell damage.

**Figure 1:**
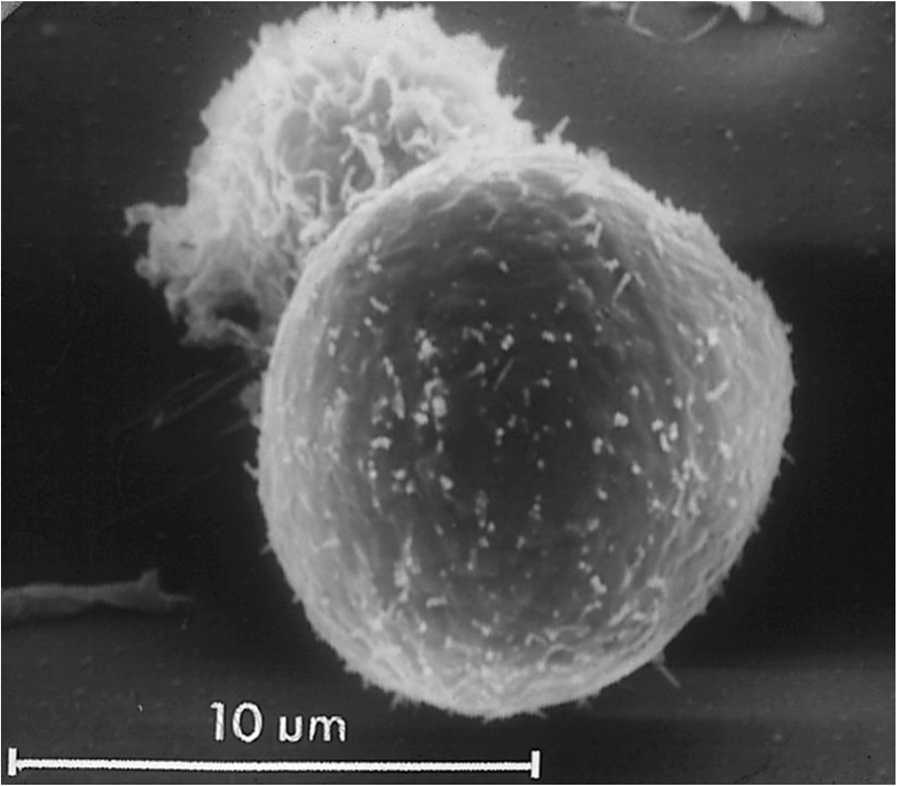
SEM picture of Alveolar Macrophages in lung lavage of a rat 24 hrs after instillation of 10.3 μm polystyrene spheres: AM in foreground with 1 phagocytized sphere showing no clearance; normal functional AM with ruffled surface in background [2].

Morrow [1] proposed the lung particle overload hypothesis based on a careful analysis of many publications on effects and disposition of particles delivered to the lungs of rodents. At the core of the particle overload hypothesis is the question about the relevancy for humans of both non-neoplastic and neoplastic effects observed specifically in rats exposed chronically to extremely high concentrations of poorly soluble particles of low acute toxicity (PSP). Morrow proposed that a continuously increasing prolongation of particle lung clearance of PSP occurs when the retained lung burden exceeds a certain threshold; he identified that this effect on particle clearance is due to an impairment of the alveolar macrophage (AM) clearance function and concluded from his analysis of the data that the impaired clearance correlates with the phagocytized volumetric loading of AM. More specifically, he suggested that the impairment starts when the average composite phagocytized volume exceeds 6% of the normal AM volume, and that complete cessation of clearance occurs when this phagocytized volume reaches 60% of the normal AM volume. Morrow proposed to test this hypothesis by administering tracer doses (below overload) of radioactively-labelled either 3 μm or 10 μm polystyrene spheres to rats, the volume of one 10 μm sphere (600 μm^3^) being equivalent to ~60% of a rat’s AM volume. The clearance of both particle sizes via *in vivo* counting of chest radioactivity over 6 months in a collimated detector system confirmed that the 10 μm particles were not cleared, whereas the 3 μm particles cleared with a rat specific normal retention T½ of 80 days [2]. Indeed, examination of lung lavage samples showed that AM had readily phagocytized the large spheres so that one sphere completely filled one AM (Figure 1).

Morrow’s pioneering studies prompted additional research into the fate of particles in the lung under normal and overload conditions; for example, an increasing interstitial access of particles associated with overload [3]; or, the finding that a decrease in the alveolar inflammatory response despite increasing doses of ultrafine TiO_2_ – as indicated by the number of inflammatory neutrophils in lung lavage samples – may be explained by a shift of the inflammation from the alveolar space to the interstitium when more than 50% of the lung deposited dose has translocated to the pulmonary interstitium [4]. Could such interstitial shift of inflammation explain results in coal miners where a large portion of very high lung burdens – consistent with overload – translocated to the interstitium so that no increase of neutrophils in lung lavage is seen. And, may this primate-specific interstitial particle sequestration compartment [5] explain that coal miners – in contrast to rats – do not have an increased risk of lung cancer under lung overload conditions?

Morrow [1] also recognized that other metrics be considered in the ongoing discussion about the “overload” phenomenon; in particular he co-authored a paper indicating that severely retarded AM clearance of test particles following sub-chronic inhalation of ultrafine TiO_2_ in rats occurred at AM phagocytized doses far below the 6% AM threshold volume, but that the phagocytized particle surface area correlated very well with the observed clearance impairment of both the ultrafine and fine TiO_2_ [6]. Even taking into account the void spaces between the agglomerated phagocytized particles in AM did not improve the correlation between retained volumetric lung burden of TiO_2_ and impaired particle clearance or pulmonary inflammation, in contrast to the adequate correlation with retained TiO_2_ surface area. Since then, the importance of the particle surface area dosemetric for particle-induced pulmonary inflammatory effects has been repeatedly shown by many investigators [7-11].

The fascination with Morrow’s theory on particle overload continues, particle overload has been at the centre of debate for over 25 years, even at times when we were discussing particles with different sizes, at much lower concentrations and exposure conditions. The overload concept has upstaged debates at many public, scientific, regulatory and personal meetings. In 1998, an international workshop by ILSI in Washington set the stage for a larger debate with two main objectives [12]: (1) provide guidance for risk assessment on the interpretation of neoplastic and non-neoplastic responses of the rat lung to PSPs; and (2) to identify important data gaps in our understanding of the lung responses of rats and other species to PSPs. The consensus of the workshop participants was:

*“Because it is still not known with certainty whether high lung burdens of PSPs can lead to lung cancer in humans via mechanisms similar to those of the rat, in the absence of mechanistic data to the contrary it must be assumed that the rat model can identify potential carcinogenic hazards to humans. Since the apparent responsiveness of the rat model at overload is dependent on coexistent chronic active inflammation and cell proliferation, at lower lung doses where chronic active inflammation and cell proliferation are not present, no lung cancer hazard is anticipated”.*

In 2000, a workshop on fibre and particle toxicology was organized by the German Permanent Senate Commission for the Investigation of Health Hazards of Chemical Compounds in the Work Area (MAK Commission) in Munich to revisit similar topics with an update on emerging biological mechanisms such as particle induced pulmonary inflammatory response, anti-oxidant protection and secondary genotoxicity of particles due to inflammation under overload conditions.

The workshop participants concluded [13] that “*the lung tumors observed in chronic rat inhalation studies with high dose PSPs … are due to a secondary genotoxicity*”, which in rats “*operates only at high doses and high levels of neutrophils*” and for “*PSP, pathology in rodents indicates that if there is no inflammation there is no fibrosis, and if there is no fibrosis, there is no cancer*”. The participants also noted that surface area of PSPs is an important dose-metric and recommended that particle surface area should be measured, along with number, mass and diameter. Concerning species differences regarding DNA repair and other defence mechanisms, the participants agreed “*that at present there is no better animal model than the rat for assessing lung cancer risk for PSP. The rat appears to be the only laboratory animal species that develops lung tumours in response to PSP and, therefore, is the most sensitive species for this endpoint*”. Still, the participants recognized that “*It is unknown whether in humans secondary PSP-induced DNA damage could occur at extremely high retained particle levels in the lung. However, this appears unlikely, since even among coal workers who have been chronically exposed to very high dust levels, rates of lung tumors are not significantly increased*”.

In the late nineties, the Medical Institute for Environmental Hygiene (MIU) in Düsseldorf, completed a lifetime animal study with 19 different particles at different doses started by the late Friedrich Pott and co-workers [10,14,15]. In this study a series of particles were administered at high doses to rats by intratracheal instillation, to apply and prove the Morrow-volumetric overload hypothesis and at the same time determine induction of lung cancer by these particles. The work has initiated a lot of discussion and debate with basically two views: people that supported the “*surface*” hypothesis, while the others considered the concept of “*volumetric*” overload as the prime mechanism leading to tumours. More important however, was the split in opinions regarding the relevance of the theory to humans, if so how extrapolation should occur. Both the ILSI workshop in 1998 [12] and MAK workshop in 2000 [13] were not conclusive on this topic, since data to support it were missing.

Today we still see that the above issues are not entirely solved and that the debate is ongoing. We have invited two groups, that is Jürgen Pauluhn (formerly Bayer) and a group of authors led by Peter Morfeld (Evonik, Germany) to review the literature and to summarize the current views on particle lung overload and the relevance to predict the carcinogenic potency of PSPs. Morfeld and colleagues [16] argue that the host defence mechanisms and particle retention metrics in rats are entirely different than those in humans (or even other rodents). However, this is what the German MAK committee is adapting and the result is a rather precautionary exposure limit value [17]. Morfeld et al. contend that the classification depends highly on debatable assumptions and calculations on lung particle overload by software which is not freely available as it is for the Multiple Path Particle Dosimetry model. They argue that the total surface area of particles retained in the lung is the more appropriate metric for the inflammation and impaired clearance function. On the other hand, Pauluhn in his paper [18] argues that the existing information on mechanistic information, primarily lung inflammation and particle retention kinetics, is by and large driven by the volumetric particle dose and the AM pool, because this determines the percentage of macrophage volume that is displaced by particles leading to overload and associated impaired clearance function. However, Pauluhn also acknowledges that surface area and in particular surface chemistry and associated potential for particle dissolution play a role in the development of host responses so that volume may not be the only critical parameter that determines the likelihood of a carcinogenic potential of PSPs.

Both Pauluhn [18] and Morfeld *et al*. [16] cite the papers by Cullen et al. [19] and Tran *et al.* [7] describing effects, deposition and retention kinetics and lung lavage neutrophil data of inhaled BaSO_4_ and TiO_2_ particles in rats, and came to divergent conclusions. Morfeld et al. [16] concurred with the conclusion of the authors that particle surface area rather than volume or mass was the appropriate metric to confirm that both particle types are PSP particles; whereas Pauluhn [18] pointed to an enhanced dissolution of BaSO_4_ when fitting the data of the accumulation phase. Indeed a recent study [20] in rats with inhalation and intratracheal instillation of nano-sized BaSO_4_ reported that 95% of the retained lung burden of BaSO_4_ was cleared within 34 days post-inhalation and that the retention halftime of instilled BaSO_4_ was less than 10 days. This is significantly faster than a normal T½ of 60–80 days for both modes of BaSO_4_ delivery to the lung. Is this consistent with a PSP (poorly soluble) category for BaSO_4_? Whatever the final judgement on the studies by Cullen et al. [19] and Tran et al. [7] is, these studies provide an important contribution to the overload discussion in terms of not only viewing accumulation and retention kinetics of the inhaled particles but also determining in vivo particle dissolution rates. The result of Konduru et al. [20] reflects either an efficient rapid translocation of the 20 nm BaSO_4_ particles, or a high dissolution rate. Clearly, studies aimed at generating new insights regarding the fate of phagocytized particles in AM of rodents and primates are required to advance our thinking about the 25-year old phenomenon of lung particle overload.

After reading both invited reviews, some conclusions and questions can be summarized as:

- Before lung particle overload was brought to the attention of the scientific community by the late professor Morrow, Davies [21] had concluded from studying dust accumulation and retention in the lungs of coal miners that an observed greatly enhanced rate of accumulation *“must be associated with an increased likelihood of a particle being retained in the lungs after it has been deposited upon the surface of an alveolus”.*
- Impaired alveolar macrophage clearance function for particles remains the hallmark of lung particle overload. The originally proposed mechanism for such impaired clearance in terms of volumetric loading of AM still seems to hold for larger particles; however, the particle surface area dose-metric is a valid alternative, especially when nanoparticles are involved, knowing that surface properties and chemistry can make a significant difference in terms of particle-cell interaction which is not associated with simply the volume of the particle in question [22-25].
- The dissolution rate of the phagocytized particles inside a phagolysosome is an important determinant of the particulate state; regardless as to whether the volume or surface area is considered as dose-metric. Also, significant differences between rodents and primates regarding interstitial particle sequestration and associated retention kinetics need to be considered.
- Regarding extrapolation modelling of results from rat overload studies to human exposure conditions - some of the open questions to be discussed are: should the normalization of retained lung burdens preferably be based on the denominator alveolar surface area, lung weight, or AM number?
- Which value for alveolar surface area should be used: Surface area at total lung capacity (TLC), at functional residual capacity (FRC)? Obviously, normal breathing occurs at FRC + tidal volume (TV), so the average surface area for particle deposition to consider during breathing is FRC + ½ TV.
- Is dosimetric extrapolation modelling of results from long term particle inhalation studies in rats meaningful? A fundamental question is, which retention T½ for humans should be used? Options based on Gregoratto (2010) might be: clearance from the alveolar space to the mucociliary escalator (T½ = 400 days, for 60% of alveolar deposit), or clearance from the alveolar space to the interstitium (T½ = 700 days, for 40% of alveolar deposits), or combined clearance from the alveolar space to the mucociliary escalator and interstitium (250 days, for 100% of alveolar deposits) as sequestration compartment
- What is the biologically effective/available surface area? BET surface area is generally used as a surrogate based on studies showing good correlations with responses, however, are there better alternatives? Might the specific surface reactivity be a more relevant metric, expressed as response per unit particle surface area, and useful for purposes of hazard ranking (Rushton et al. [26]).
- Is there evidence for particle overload associated impaired clearance in humans? There seems to be conditional evidence in coal miners as indicated by Davies [21] and based on calculations by Stöber et al. [27] using autopsied lung samples from miners and from actual magneto-pneumographic measurements of dust retention in coal miners’ lungs by Freedman and Robinson [28].

In a time when kinetics and overload belong to old school descriptive toxicology, we felt it is our obligation to bring the discussion back to the scientific forum and out of the risk assessment arena. In addition, for our younger readers who are mainly focused on molecular mechanistic endpoints within specific area’s such as the inflammasome, DNA repair or oncogene-regulation, this may also be a message that findings need to be viewed and interpreted in a translational context. Driven by the new events such as SCOEL using the overload approach using a volumetric approach and defining elimination kinetics to half-times exceeding 60-days, and AM pool size as the key elements] for setting occupational exposure levels in the EU, the editor of PFT decided to invite key players in the field to give their opinion on the most important events of the past 10–15 years, and give their opinion on dose-metrics and rat-human extrapolation. Both the submission and reviewing process showed how topical the overload issue still is, including the impact and debate on its use for risk assessment. At the same time it is interesting to note that some toxicologists don’t even realize what the debate is all about. We hope that with this mini-series of 2 papers [16,18] we have positioned the debate in the current time frame. We believe this enables the arguments to be separated from the political arena, allowing a renewed objective debate. This procedure goes back to the roots and mission of the founding of our open-access journal, dedicated to general aspects of particle and fibre toxicology [29].
